# Pandemic-related challenges accessing food and primary healthcare among sex workers during the COVID-19 pandemic: findings from a community-based cohort in Vancouver, Canada

**DOI:** 10.1186/s12889-024-18959-z

**Published:** 2024-06-07

**Authors:** Elizabeth Frost, Kate Shannon, Melissa Braschel, Mary Kestler, Jennie Pearson, Chelsey Perry, Shira M. Goldenberg

**Affiliations:** 1 Joint Doctoral Program in Public Health, San Diego State University and UC San Diego, San Diego, USA; 2 Center for Gender and Sexual Health Equity, University of British Columbia Faculty of Medicine, Vancouver, Canada; 3 Division of Social Medicine, University of British Columbia, Vancouver, Canada; 4 Oak Tree Clinic, BC Women’s Hospital, Vancouver, Canada; 5 Division of Epidemiology and Biostatistics, School of Public Health, San Diego State University, San Diego, USA

**Keywords:** Food access, Healthcare access, Economic vulnerability, Sex workers, COVID-19

## Abstract

**Introduction:**

Globally, the COVID-19 pandemic upended healthcare services and created economic vulnerability for many. Criminalization of sex work meant sex workers were largely ineligible for Canada’s government-based financial pandemic relief, the Canadian Emergency Response Benefit. Sex workers’ loss of income and inability to access financial support services during the pandemic resulted in many unable to pay rent or mortgage, and in need of assistance with basic needs items including food. Little is known about the unique experiences of sex workers who faced challenges in accessing food during the pandemic and its impact on healthcare access. Thus, we aimed to identify the association between pandemic-related challenges accessing food and primary healthcare among sex workers.

**Methods:**

Prospective data were drawn from a cohort of women sex workers in Vancouver, Canada (An Evaluation of Sex Workers’ Health Access, AESHA; 2010-present). Data were collected via questionnaires administered bi-annually from October 2020-August 2021. We used univariate and multivariable logistic regression with generalized estimating equations to assess the association between pandemic-related challenges accessing food and challenges accessing primary healthcare over the study period.

**Results:**

Of 170 participants, 41% experienced pandemic-related challenges in accessing food and 26% reported challenges accessing healthcare. Median age was 45 years (IQR:36–53), 56% were of Indigenous ancestry, 86% experienced intimate partner violence in the last six months, and 62% reported non-injection substance use in the last six months. Experiencing pandemic-related challenges accessing food was positively associated with challenges accessing primary healthcare (Adjusted Odds Ratio: 1.99, 95% Confidence Interval: 1.02–3.88) after adjustment for confounders.

**Conclusions:**

Findings provide insight about the potential role community-based healthcare delivery settings (e.g., community clinics) can play in ameliorating access to basic needs such as food among those who are highly marginalized. Future pandemic response efforts should also take the most marginalized populations’ needs into consideration by establishing strategies to ensure continuity of essential services providing food and other basic needs. Lastly, policies are needed establishing basic income support and improve access to food resources for marginalized women in times of crisis.

## Introduction

Globally, sex workers represent an underserved and highly marginalized population that face a disproportionate burden of HIV and sexually transmitted infections resulting from structural oppression [1, 2]. Criminalization, discrimination from healthcare providers, stigma, and violence towards sex workers have been shown to contribute to these health inequities and impede access to needed healthcare services [3–6]. While interruptions and vulnerabilities engendered by the COVID-19 pandemic is postulated to have amplified these health inequities and created widespread negative impacts on health, social, and economic outcomes for sex workers [5, 7], there remains a dearth of empirical quantitative evidence examining this, particularly in a high-income country like Canada where universal healthcare and supportive social services exists but are inaccessible for many marginalized individuals.

During the height of the COVID-19 pandemic, interruptions to services accessed by sex workers included closures of community-based health clinics resulting in restricted access to prescriptions, gynecological exams, and other reproductive health services [8, 9]. Despite options for telemedicine appointments, many sex workers reported barriers to accessing virtual healthcare services due to a lack of phone, internet, or Wi-Fi [5]. As with many other precarious and informal workers, sex workers faced high risk of COVID-19, exacerbated by the close-contact nature of their profession and lack of access to occupational protections and economic benefits [5, 10]. For example, a recent community report of Canadian sex workers found only 45% of sex workers were able to access masks in the first year of the pandemic [8]. Additionally, sex workers encounter higher levels of stigma and discrimination compared to other populations when trying to access healthcare facilities, which pose serious barriers to receiving needed medical care [5, 11, 12].

In countries such as Canada where primary healthcare services are publicly funded for most of the population and costs of prescription drugs are covered for low-income individuals, healthcare utilization for socially and economically marginalized populations can still be impacted by economic-related structural barriers. Sex workers, like many other marginalized populations, may struggle with paying for transportation to the doctor’s office, and experience reduced earnings from taking the time to attend a medical appointment, thus further preventing them from receiving needed primary healthcare services. For someone who is already economically marginalized, the need to work and make an income may be a higher priority compared with attending a doctor’s visit.

Nation-wide restrictions on in-person contact in Canada and closures of formal venue-based sex work establishments such as massage parlors, micro-brothels, and hotels negatively impacted sex workers by exacerbating economic vulnerability, leaving many sex workers with little means to support themselves financially [13]. Criminalization of sex work meant sex workers were largely ineligible for Canada’s government-based financial pandemic relief, Canadian Emergency Response Benefit (CERB) [5, 8, 14]. Sex workers’ loss of income and inability to access financial support services during the pandemic resulted in many unable to pay rent or mortgage, and in need of assistance with basic needs items including food [5, 7, 8, 13, 15].

In Canada, sex workers faced high levels of food insecurity pre-pandemic (74%) [16]. During the first year of the COVID-19 pandemic, community reports predicted that two-thirds of Canadian sex workers were receiving food or meals from community-based organizations and food pantries [5]. However, due to stigma by association, Canadian social service organizations that worked directly with the sex work community encountered challenges in adequately securing funding for needed social and health services to assist sex workers [5]. Despite evidence pointing to the high prevalence of food insecurity among sex workers, particularly at the time of the COVID-19 pandemic, few studies have examined the specific challenges sex workers encountered in accessing food during the pandemic when Canada first mandated lockdowns and restrictions. There is a gap in understanding the unique experiences of sex workers navigating obtaining basic needs such as food and healthcare services amid COVID-19 lockdowns.

This study aimed to identify the association between experiencing pandemic-related challenges accessing food and challenges accessing primary healthcare during the first year of the COVID-19 pandemic in a community-based cohort of sex workers in Metro Vancouver, Canada.

## Methods

### Study design

Data were drawn from an ongoing community-based cohort, An Evaluation of Sex Workers Health Access (AESHA) which initiated recruitment in 2010. AESHA collaborates with sex worker community agencies in the Metro Vancouver area and is overseen by a community advisory board of sex work organizations and community members. Inclusion criteria includes identifying as a woman (including transgender women), having exchanged sex for money within the past 30 days, and providing written informed consent to participate. AESHA participants were recruited by a frontline team of community-based staff members using time-location sampling, a method using daytime and late-night recruitment with community mapping to identify indoor (i.e., massage parlors, micro-brothels, hotels, bars) and outdoor (i.e., streets, alleys) sex work solicitation locations, as well as online solicitation spaces. Further detail on AESHA is available elsewhere [17].

This study used longitudinal data from the main AESHA interviewer administered questionnaire including responses on socio-demographics and individual level characteristics. It also used a supplementary COVID-19 questionnaire implemented bi-annually from October 2020-August 2021 that examined pandemic impacts on housing and economic factors; work environment; safety, violence, and policing; and social outcomes. Based on early community feedback during the pandemic, measures assessed on the COVID-19 questionnaire were developed to capture the impact of the pandemic on sex workers – a uniquely marginalized group – and to understand their unique experiences with regards to a range of hypothesized health, social, and economic impacts during that first year during the pandemic, when access to food outlets and other resources was restricted in a variety of ways for many marginalized groups locally.

Given the challenges of connecting with sex workers during COVID-19 lockdowns, closures, and service disruptions, the research team was able to reach a relatively small sample of the AESHA cohort, which may have resulted in some temporal variation. The COVID-19 questionnaire was administered by phone or in-person based on institutional and public health guidelines for research during various stages of the pandemic response. Participants received $45 CAD for each visit for the main questionnaire and an additional $20 CAD for the COVID-19 supplementary questionnaire. Ethics approval was obtained from the Providence Health Care/ University of British Columbia Research Ethics Board.

### Outcome

Pandemic-related challenges accessing primary healthcare was assessed by the question, “In the last 6 months, have you experienced difficulty accessing a family doctor, nurse or clinic for routine healthcare or prescriptions for any of the following reasons since the COVID-19 pandemic began in March, 2020?” Response options included: canceled or reduced services; inability or lack of knowledge on how to access telehealth or other virtual/phone/online services; family doctor, nurse or clinic didn’t offer virtual/phone/online services; less available time to visit doctor, nurse or clinic; afraid of using services for fear of sick; or relevant other responses. Participants were directed to select all response options that applied to their situation or to select none if they did not experience any of these challenges. Those who responded “Yes” to any of the response options were coded as “Yes” and all others were categorized as “No”.

### Primary exposure

Pandemic-related challenges accessing food during the pandemic was assessed by the question, “In the last 6 months, have you experienced any of the following changes related to any new or ongoing impacts since the COVID-19 pandemic began in March 2020?” This measure was selected for analysis as the question and response options were developed to capture sex workers’ lived experiences in the local context during the early phase of the pandemic. Response choices included: Afraid to leave house for food due to fear of getting sick; Reduced or no supply of food at place you buy food; prices higher at place you buy food; Stores you access food at are closed, have limited hours or lines are too long; You experienced problems with online food delivery; The restaurant where you normally access food is now closed, has limited hours or lines are too long; You had difficulty meeting new food bank registration requirements; Service center where you normally access food is closed, has limited hours or long lines; Food provided in your housing establishment has been reduced or cancelled (e.g., reduced from two meals down to one meal or meals on alternative days); or relevant other responses describing food access. Participants were directed to select all response options that applied to their situation or none if they did not experience any of these issues. Participants who selected any of the response categories were categorized as “Yes” and all others were categorized as “No”.

### Other study variables

Socio-demographic and structural level variables were selected a priori from literature on food access and sex workers’ health. Socio-demographic factors included: age; Indigenous ancestry (First Nation, Inuit or Metis); sexual minority (lesbian, gay, bisexual, asexual, queer, Two-Spirit or other); gender minority (transgender, transsexual, non-binary, genderqueer, Two-Spirit or other); lifetime mental health diagnosis (depression, anxiety, post-traumatic stress disorder-PTSD, Schizophrenia or Schizoaffective, bipolar disorder or relevant other diagnosis); living with HIV (confirmed seropositivity); reporting self-rated good health (self-reported excellent, very good or good health status); and substance use in the last six months (both injection and non-injection, excluding alcohol and cannabis). Structural level variables impacting the participants at a systems level included: negative changes in housing security in the last six months (difficulty finding a safe place to stay, being relocated to temporary housing, living with more people, less privacy or personal space); experiencing homelessness or living on the streets in last six months; currently cohabiting (always or usually living with other people); changes to non-sex work employment in last six months (laid off, voluntarily quit job due to health concerns, forced to work fewer hours or shifts, reduction in wages, unemployment); lifetime experience of community violence (verbally harassed, threatened or physical violence by community resident or business owner); intimate partner violence (IPV) in the past six months (experienced any physical, sexual or emotional violence from a male partner) measured by an abridged version of the WHO Standardized IPV Scale Version 9.9 [18, 19]; and any physical or sexual violence by perpetrators including clients, intimate partners or others in past six months.

The primary exposure and outcome variables were time-updated to reflect occurrences from the past six months. Other variables of interest and confounders included in the analysis were also time-updated, with the exception of Indigenous ancestry, gender identity, and sexual orientation which were considered time-fixed variables.

### Statistical analysis

Analyses were conducted among 170 AESHA participants between October 2020-August 2021 who answered the supplementary COVID-19 questionnaire. Descriptive statistics were calculated for socio-demographic and structural confounders, the primary exposure (pandemic-related challenges accessing food), and the primary outcome (pandemic-related challenges accessing primary). Frequencies and proportions were calculated for categorical variables and measures of central tendencies (median and interquartile range (IQR)) for continuous variables.

Variables were stratified by difficulty accessing primary healthcare and compared using Pearson’s chi square test for all categorical variables (or Fisher’s exact test where cell counts were small) and Wilcoxon rank sum test for continuous variables. Bivariate and multivariable analysis used logistic regression to assess associations with challenges accessing primary healthcare. Generalized estimating equations (GEE) were used to account for repeated measures among participants over time and used an exchangeable correlation structure. Using a complete case approach to handle missing data, GEE analysis was further restricted to 151 participants (209 observations) after removing 19 participants with 38 observations (loss to follow-up: 11.1% (*n* = 19/170)). Multivariable analysis of the association between pandemic-related challenges accessing food and challenges accessing primary healthcare examined a priori hypothesized confounders that were selected based on previous sex work research literature, which appeared to be associated with the outcome in univariate analysis, and which had complete data available. Statistical analyses were performed in SAS version 9.4 (SAS, Cary, NC), and all p-values are two-sided.

## Results

### Socio-demographic and structural characteristics

Among 170 women sex workers, the median age was 45 years old. Over half (56%, *n* = 96) of participants identified as Indigenous, 57% (*n* = 97) had self-rated good health, 80% (*n* = 136) were diagnosed with a mental health issue, and 62% (*n* = 106) reported non-injection substance use in the last six months at baseline (Table 1). The prevalence of intimate partner violence was 86% (*n* = 147), lifetime community violence was 51% (*n* = 86), and recent (in the last six months) physical/sexual violence by clients, intimate partners or others was 23% (*n* = 39). During the study period, 38% (*n* = 65) of participants reported currently cohabitating, 9% (*n* = 16) were homeless in the last six months, and 10% (*n* = 17) who experienced changes in housing security in the last six months at baseline. When asked about changes to non-sex work employment in the last six months at baseline, 18% (*n* = 31) of participants stated they were either laid off, had quit, worked fewer hours, had reduced wages or were currently unemployed.

**Table 1:** Baseline characteristics stratified by COVID-19 pandemic-related challenges accessing primary care in the last six months among women sex workers in Vancouver, Canada, AESHA, October 2020-August 2021 (*N* = 170)

Characteristic	Experienced pandemic-related challenges accessing primary care**			
		Yes (%) *n* = 44	No (%) *n* = 126	*p* – value***
**Individual factors**				
Age (median, IQR)	45 (36–53)	44 (34–53)	46 (37–53)	0.335
Indigenous ancestry	96 (56.5)	21 (47.7)	75 (59.5)	0.174
Sexual minority	93 (54.7)	28 (63.6)	65 (51.6)	0.167
Gender minority	32 (18.8)	8 (18.2)	24 (19.1)	0.899
Mental health diagnosis, ever	136 (80.0)	35 (79.6)	101 (80.2)	0.930
Self-rated good health**	97 (57.1)	24 (54.6)	73 (57.9)	0.618
Living with HIV	26 (15.3)	7 (15.9)	19 (15.1)	0.895
Non-injection substance use*	106 (62.4)	32 (72.7)	74 (58.7)	0.074
Injection substance use	49 (28.8)	13 (29.6)	36 (28.6)	0.859
**Structural factors**				
Pandemic-related challenges accessing food**	70 (41.2)	25 (56.8)	45 (35.7)	0.014
Pandemic-related changes to housing security**	17 (10.0)	3 (6.8)	14 (11.1)	0.564
Homeless**	16 (9.4)	4 (9.1)	12 (9.5)	1.00
Currently cohabiting	65 (38.2)	20 (45.5)	45 (35.7)	0.284
Pandemic-related changes to non-sex work employment**	31 (18.2)	10 (22.7)	21 (16.7)	0.370
Experienced community violence, ever	86 (50.6)	26 (59.1)	60 (47.6)	0.190
Experienced intimate partner violence**	22 (12.8)	9 (20.0)	13 (10.2)	0.097
Experienced physical or sexual violence**	39 (22.9)	1515 (34.1)	24 (19.1)	0.074

* Excluding alcohol and cannabis.

** In the past six months.

***P-value is provided for the Pearson’s chi square test for all categorical variables (or Fisher’s exact test where cell counts were small) and Wilcoxon rank sum test for continuous variables.

### Pandemic-related challenges accessing food

A total of 41% participants reported challenges accessing food during the pandemic. Participants’ responses show that 17.7% were afraid to leave the house for food due to fear of getting sick (*n* = 30), 11.2% had reduced or no supply of food at place they bought food (*n* = 19), 18.8% experienced prices higher at place they bought food (*n* = 32), 9.4% reported stores they usually accessed food at were closed, had limited hours or lines are too long (*n* = 16), and 5.3% reported that food provided in housing had been reduced or cancelled (*n* = 16).

### Multivariable analysis of pandemic-related challenges accessing food and challenges accessing primary healthcare

A total of 26% participants reported challenges accessing primary healthcare. In GEE analysis (Table 2), the unadjusted odds of experiencing challenges accessing healthcare were significantly higher among participants who reported pandemic-related challenges accessing food (Odds Ratio (OR) 1.99, 95% Confidence Interval (CI) 1.05–3.78), and among those who experienced changes in non-sex work employment (OR 2.02, 95% CI 1.00-4.07) in the last six months.

**Table 2:** Bivariate analysis of COVID-19 pandemic-related challenges accessing primary healthcare among sex workers in Vancouver, AESHA, October 2020-August 2021 (*N* = 151)1

Characteristic (Yes vs. No)	Unadjusted Odds Ratio (95% CI)
**Individual Factors**	
Age *(per year older)*	0.98 (0.95–1.01)
Indigenous ancestry (yes vs. no)	0.62 (0.34–1.13)
Sexual minority (yes vs. no)	1.44 (0.78–2.65)
Gender minority (yes vs. no)	1.31 (0.65–2.66)
Ever had mental health diagnosis (yes vs. no)	0.90 (0.44–1.82)
Self-rated good health (yes vs. no)	0.85 (0.48–1.51)
Living with HIV (yes vs. no)	0.99 (0.43–2.28)
Non-injection substance use^2,3^ (yes vs. no)	1.92 (0.97–3.78)
Injection substance use^3^	1.11 (0.60–2.04)
**Structural Factors**	
Pandemic-related challenges in accessing food^2^ (yes vs. no)	1.99 (1.05–3.78)
Pandemic-related changes to housing security^2^ (yes vs. no)	0.79 (0.25–2.52)
Homeless^3^ (yes vs. no)	0.67 (0.23–1.95)
Currently cohabiting (yes vs. no)	1.55 (0.84–2.85)
Pandemic-related changes to non-sex work employment^2^ (yes vs. no)	2.02 (1.00–4.07)
Ever experienced community violence (yes vs. no)	1.38 (0.75–2.54)

1. GEE analysis included 209 observations.

2. Excluding alcohol and cannabis.

3. Time updated measure capturing events in the last six months.

In multivariable GEE analysis (Table 3), pandemic-related challenges accessing food among women sex workers was associated with challenges accessing primary healthcare during the COVID-19 pandemic (Adjusted Odds Ratio (AOR) 1.99, 95% CI 1.02–3.88) after adjusting for Indigenous ancestry, age, sexual minority, and non-injection substance use.

**Table 3:** Multivariable model of the association between COVID-19 pandemic-related challenges in accessing food and pandemic-related challenges accessing primary healthcare among sex workers in Vancouver, AESHA, October 2020-August 2021 (*N* = 151)

Characteristic	Unadjusted Odds Ratio (95% Confidence Interval)	Adjusted Odds Ratio* (95% Confidence Interval)	
Pandemic-related challenges in accessing food (yes vs. no)	1.99 (1.05–3.78)	1.99 (1.02–3.88)	

*Model adjusted for Indigenous ancestry, age, sexual minority, and non-injection substance use (excluding alcohol and cannabis) in past six months.

** Time updated measure capturing events in the last six months.

## Discussion

This study examined self-reported changes brought upon by the COVID-19 pandemic in accessing food and primary healthcare in a highly marginalized occupational cohort of women sex workers in Vancouver, Canada between 2020 and 2021. Almost half of sex workers in this study interviewed reported challenges accessing food and over a quarter reported challenges accessing primary healthcare during the COVID-19 pandemic. After adjusting for confounders, we found that experiencing pandemic-related challenges accessing food was associated with twofold higher odds of experiencing challenges accessing primary healthcare during the COVID-19 pandemic.

Our study expands upon previous knowledge about food insecurity by exploring issues related to food access (i.e., where and how sex workers obtain food) during the pandemic and associated public health response measures. Despite a purportedly universal healthcare system, marginalized individuals in Canada face numerous structural barriers to food and healthcare access [20]. Previous studies have established that the pandemic disproportionately impacted those most vulnerable in the community [21, 22]. This includes sex workers who were found to have experienced economic instability and financial difficulties due to reduced employment and pandemic related closures or restrictions which in turn led to reduced income and inability to pay for food or housing [23]. As noted in our study, the ability to afford food was only one dimension of how the pandemic impacted sex workers’ food access. Pandemic-related closure of restaurants, limited hours of grocery stores and long lines, and limited public and community services such as food banks or food services offered in community-based organizations (e.g., housing) were also reported by sex workers. These challenges were likely compounded by structural factors during the pandemic including increased stigma and discrimination, violence, housing discrimination, and inability to access government financial relief [5, 11, 14, 23–25].

This study builds on previous research regarding the relationship between food and health care access, and provides unique insights that are of critical importance for supporting pandemic preparedness and advancing health equity for sex workers. To our knowledge, it is the first to examine the prevalence or correlates of food access or its relationship to healthcare access among sex workers during the COVID-19 pandemic in Canada or other similar high-income settings. This research is of critical importance as sex workers faced severe structural marginalization pre-pandemic, including discrimination and stigma from business owners, community members, and medical community [11, 26, 27]. Broader research suggests bidirectional links between food insecurity and healthcare utilization and access, with prior studies documenting both positive and negative associations [28, 29]. However, much of the research evaluating food insecurity during the start of the pandemic may not have had a high representation of the most marginalized groups like sex workers. Sex workers face well-documented pre-pandemic health and social inequities, including those related to HIV, substance use, food insecurity, sexual health care, and serious institutional barriers to healthcare; during the pandemic, many were unable to stop working and faced exposure risks in the context of close contact work and lack of occupational protections and access to PPE [2, 5, 8, 10, 30]. Furthermore, previous literature demonstrates that sex workers experienced severe economic hardships and had limited access to economic benefits due to their precarious and marginalizedstatus [14].

Findings from this study can help in developing policies and services that better address the health and well-being of sex workers. We recommend establishment of basic income support for sex workers to meet their needs for food, housing, and other needs. In addition, interventions are recommended in community-based healthcare delivery settings (e.g., community clinics) – for example, integrating food pantries within healthcare systems could help address health and food needs at the same time, and community-based programs that screen patients for food insecurity and provide meals and other food items should be scaled-up [31]. We also recommend that future pandemic response efforts take the most marginalized populations’ needs into consideration by establishing strategies to ensure continuity of essential services providing food and other basic needs. Lastly, we recommend that sex work be fully decriminalized as a longer-term structural change associated with reduced stigma and increased access to health and social services, and which could reduce structural marginalization including accessing to basic needs by reducing discrimination and increasing economic options and agency for sex workers [2, 32, 33].

This study relies on self-reported questionnaire data, which may be subject to recall and social-desirability bias that could have resulted in under-reporting of stigmatized or sensitive information. However, our frontline staff are highly trained in non-stigmatizing and trauma-informed interviewing and have lived experiences in sex work and strong community ties to the Vancouver area, all of which helped mitigate this. This study was conducted among a relatively small sample of participants whom we were able to reach during the first year of the pandemic when many indoor sex work venues were closed and in-person research activities curtailed. Our sample over-represents sex workers who are white, of Indigenous ancestry and worked in outdoor settings and informal sex work venues. The overrepresentation of Indigenous women is rooted in the impact of historical and current colonial structures that lead to a significant number of Indigenous women working in street-based and public sex work [34]. It is also a reflection of the disproportionate levels of policing, criminalization, racism, and discrimination faced by Indigenous sex workers and women [35]. Non-Indigenous women of color as well as im/migrant sex workers and those working in formal indoor venues (e.g., massage parlors) are under-represented in our sub-sample, due to limitations in conducting in-person research in these work environments during the pandemic. Further research is needed to fully elucidate the unique experiences of diverse groups of sex workers, including Black, Asian and Latinx sex workers.

This study used data from the main semi-annual AESHA questionnaire and a COVID-19 supplementary questionnaire administered with the same participants. A small number of questionnaires were completed up to three months apart, which may result in some temporal variation. In addition, this analysis used unique, community-developed measures of pandemic-related disruptions in food access and primary care, as our interactions with the community during the early months of the pandemic suggested that more specific questions asking about impacts of the COVID-19 pandemic were needed to fully capture its impacts with this population (as opposed to comparing pre/post COVID-19 data using less specific measures, such as a food insecurity scale). Thus, while a strength of the study lies in its community-informed measure addressing local contextual experiences in challenges accessing food among sex workers during the pandemic, a limitation is that this is not directly comparable with standardized measures of food insecurity used in broader contexts. Further, it is possible that unmeasured variables that may confound the relationship between access to food and healthcare were not assessed; however, AESHA uses an extensive survey to mitigate this, and the analysis carefully considered all available potential confounders hypothesized to be related to access to food and healthcare in this population, thus we believe this to be unlikely.

## Conclusion

In summary, the association between pandemic-related challenges in accessing food and challenges accessing primary healthcare among sex workers highlight the need to dismantle structural barriers inhibiting access to healthcare and social services, including sex work criminalization and occupational stigma. Knowledge gained from this study can help guide policies supporting equitable access to healthcare and income supports to cover basic needs, including food, for marginalized women during and beyond the pandemic.

## Data Availability

Due to our ethical and legal requirements related to protecting participant privacy and current ethical institutional approvals, de-identified data are available upon request pending ethical approval. Please submit all request to initiate the data access process to the corresponding author.

## Abbreviations

AESHA: An Evaluation of Sex Workers’ Health Access
IPV: Intimate partner violence
